# Severe Penile Ischemia After Metallic Ring Strangulation: A Rare Case of Complete Functional Recovery

**DOI:** 10.7759/cureus.90406

**Published:** 2025-08-18

**Authors:** Manuel M Lopes, Vasco Quaresma, João Lorigo, Pedro Nunes, Arnaldo Figueiredo

**Affiliations:** 1 Urology and Renal Transplantation, Hospitais da Universidade de Coimbra, Coimbra, PRT

**Keywords:** foreign body, functional recovery, ischemia, penile strangulation, urological emergency

## Abstract

Penile strangulation by constricting devices is a rare but potentially devastating urological emergency. The removal of such foreign bodies usually poses significant challenges, and late presentations may result in irreversible complications.

We report the case of a 56-year-old man who presented to the emergency department with pain and necro-ischemic penile signs due to a metallic ring placed at the base of the penis for over 48 hours. Physical examination revealed grade IV-V ischemic injury and urinary retention. The constricting body was removed using an angle grinder under anesthesia. Ischemic changes led to partial skin necrosis, requiring debridement and circumcision on day 7 after admission, followed by a split-thickness skin graft on day 20 of hospital stay with reconstructive and plastic surgery collaboration. The patient was discharged home after 28 days, with no infections or functional complications. At one-year follow-up, the patient had preserved voiding and spontaneous erectile function.

This case illustrates a multidisciplinary approach to a rare urological emergency and highlights the feasibility of unconventional yet safe surgical techniques for object removal.

## Introduction

Penile strangulation by constrictive devices is a rare but potentially devastating urological emergency that requires prompt evaluation and intervention. These devices, typically placed at the base of the penis to enhance or prolong erection, can lead to progressive venous congestion, ischemia, and eventually necrosis if not promptly removed [1,2]. First described in the 18th century, the phenomenon has been reported with increasing frequency, particularly in adult men engaging in autoerotic or recreational sexual activity [3].

A wide range of objects have been implicated, including metal rings, bottle necks, nuts, and even hammer heads [2,4]. The removal of such foreign bodies poses significant challenges due to the hardness, shape, and location of the object. Conventional instruments are often insufficient, and in some cases, multidisciplinary collaboration is required, from creative and innovative methods to the use of industrial tools [5-7].

Several grading systems have been proposed to categorize the severity of penile injury, with the scale introduced by Bhat et al. being the most referenced [8]. This five-grade system ranges from mild edema (grade I) to gangrene or amputation (grade V). Prognosis is closely tied to the duration of incarceration and the timing of intervention, with injuries beyond 48-72 hours significantly increasing the risk of high-grade complications, in terms of sexual and urinary function [1,2].

## Case presentation

**Figure 1:**
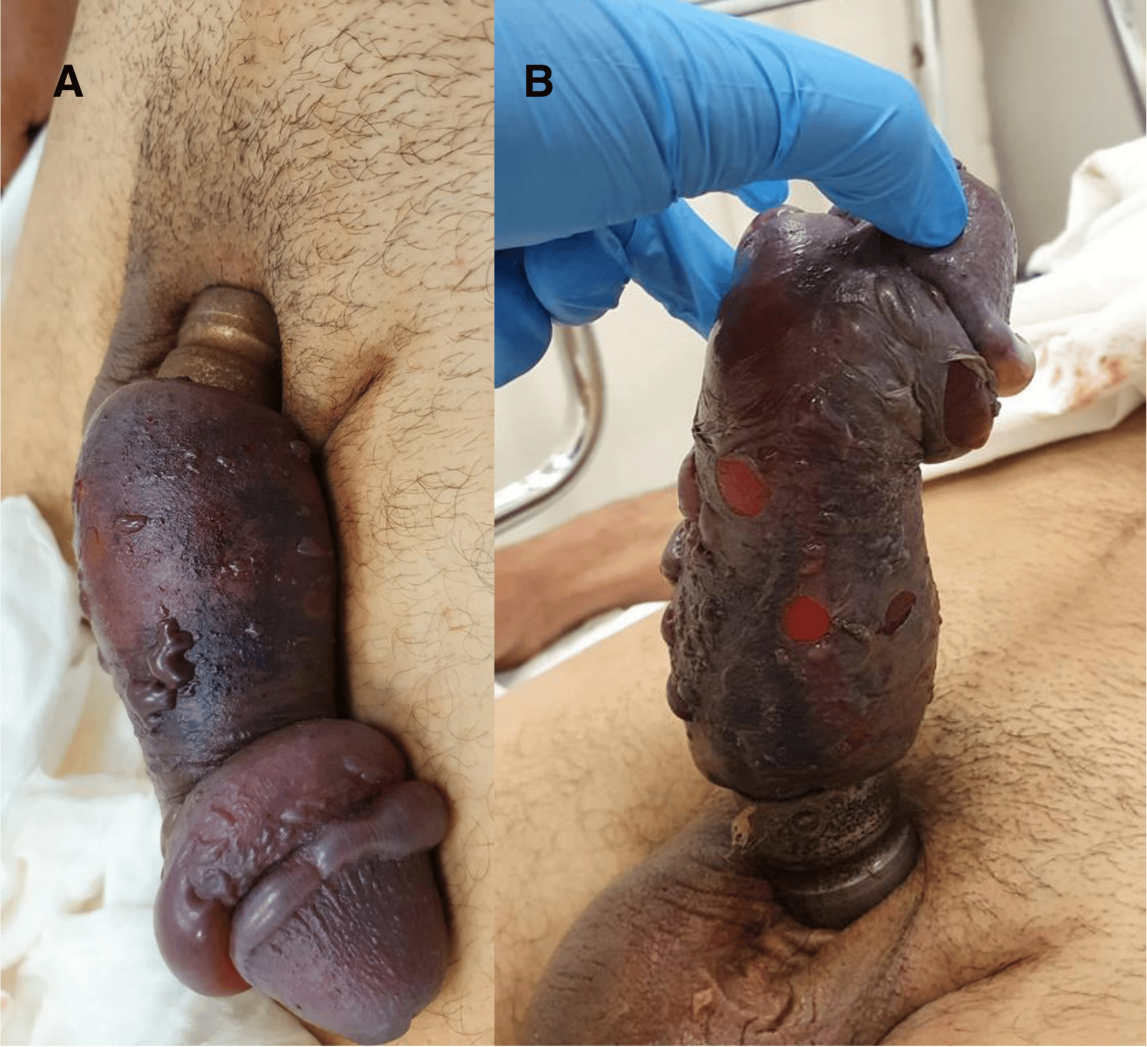
Initial presentation with ischemic signs: dorsal (A) and ventral view (B).

A 56-year-old man with no relevant past medical history presented to the emergency department complaining of penile pain and progressive swelling caused by a penile constricting ring for more than 48 hours, placed for sexual pleasure. Clinical examination revealed a tight metallic ring incarcerating the base of the penis, with marked edema and signs of severe ischemia (Figure 1): a grade IV-V penile injury according to Bhat et al.'s classification [8]. The patient was in severe pain and urinary retention for the last eight hours. No other abnormalities were noted during the initial evaluation. The patient's baseline International Index of Erectile Function-5 (IIEF-5) score was 22.

Due to the thickness and rigidity of the object, conventional cutting tools proved insufficient for its removal. Additionally, the complexity of the case and the need for specialized urological management warranted the patient's transfer to a tertiary care center. The patient was emergently taken to the operating room, where the metallic ring was successfully cut and removed using an angle grinder (Video 1). Adequate protection with moist gauze and metallic spatulas was used to avoid collateral damage, such as thermal or mechanical injury to the surrounding tissues. A suprapubic catheter was placed for bladder decompression and urinary diversion. Antibiotic prophylaxis with amoxicillin 1 g every eight hours was started in the room and continued during the first 14 days of hospital stay.

**Video 1:** Surgical management for penile ring extraction.

**Figure 2:**
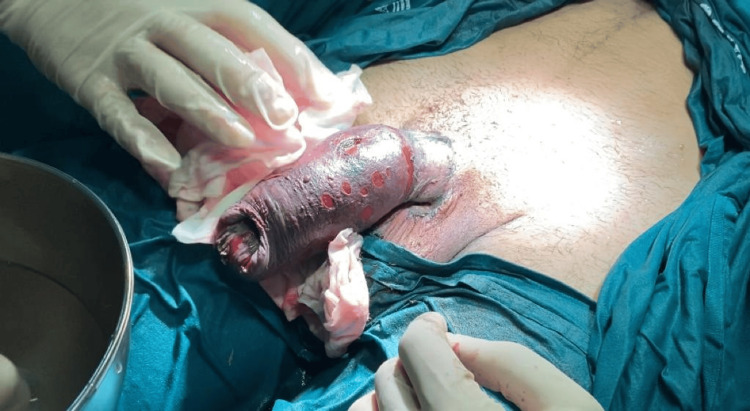
Intraoperative appearance immediately after ring removal.

The team decided on a watchful waiting strategy for further management, despite the aspect after immediate decompression (Figure 2). Following ring removal, necrotic and ischemic skin changes remained for the first two days. Daily wound care and monitoring were performed. Analgesics for pain control and tadalafil in a 5 mg daily dose for theoretical penile rehabilitation were started.

**Figure 3:**
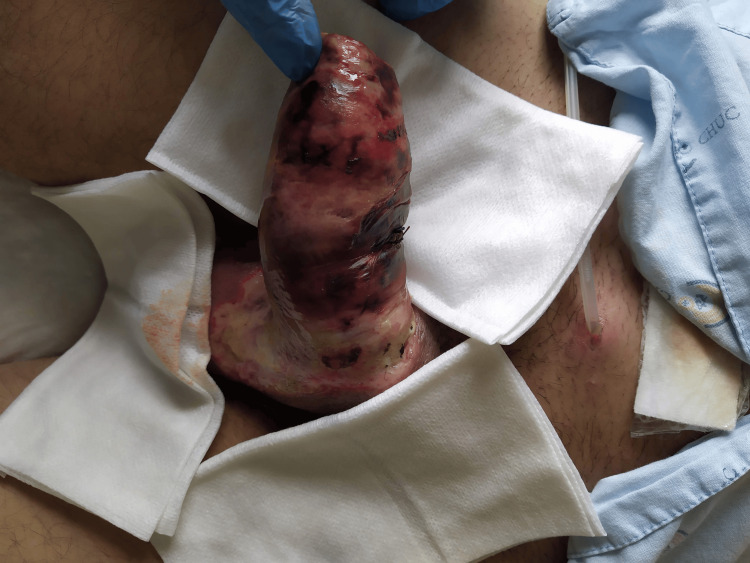
Penile appearance on day 5: necrotic changes pre-debridement.

**Figure 4:**
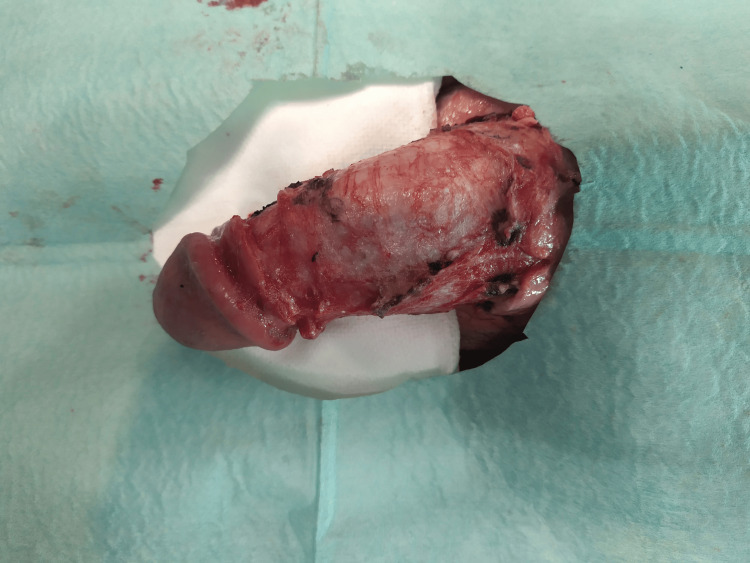
Postoperative appearance after debridement and circumcision on day 7.

After day 3, progressive signs of tissue detachment were seen (Figure 3). On day 7, necrotic skin was debrided, and a circumcision was performed. The corpora cavernosa and corpus spongiosum had a viable appearance (Figure 4). At the end of the first week, the patient reported spontaneous urethral voiding.

**Figure 5:**
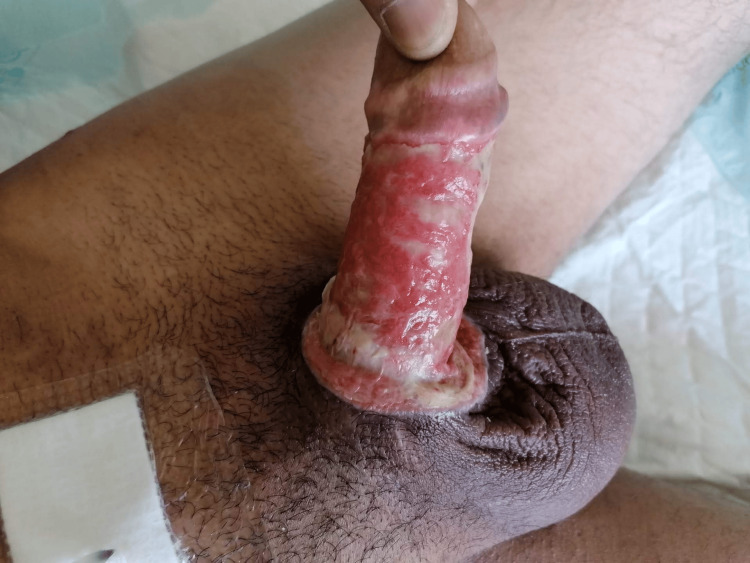
Corpora tissue aspect 15 days since admission.

During the next seven days, wound treatment and surveillance were maintained, with a good evolution of the corpora tissue (Figure 5). Although there was spontaneous voiding, the suprapubic catheter was kept in order to avoid tissue contamination with urine. The patient surprisingly reported a nocturnal erection on day 15.

**Figure 6:**
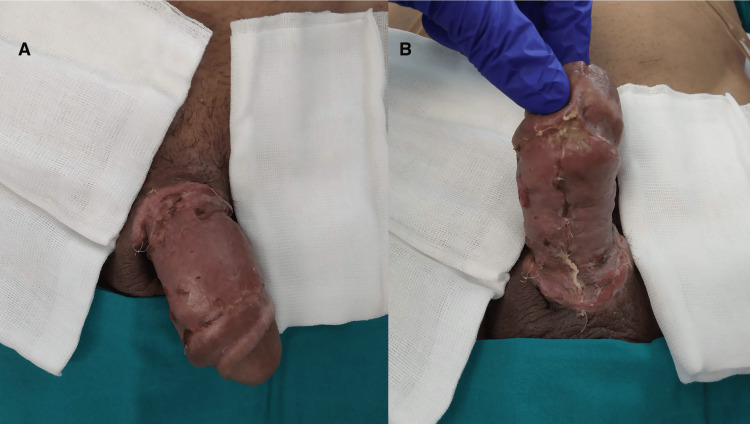
Grafted penile shaft with signs of good integration: dorsal (A) and ventral view (B).

On day 20 after presentation, in collaboration with the reconstructive and plastic surgery team, a skin graft harvested from the anterior thigh was applied to the penile shaft (Figure 6). A preoperative single administration of 2 g cefazoline was given.

The patient remained hospitalized for 28 days. The suprapubic catheter was removed the day before discharge. There were no infectious or urethral complications noticed. Graft integration was estimated at 90%. Outpatient recommendations included daily wound care, no sexual activity, and no strenuous physical activity until the next medical reevaluation, one month later.

**Figure 7:**
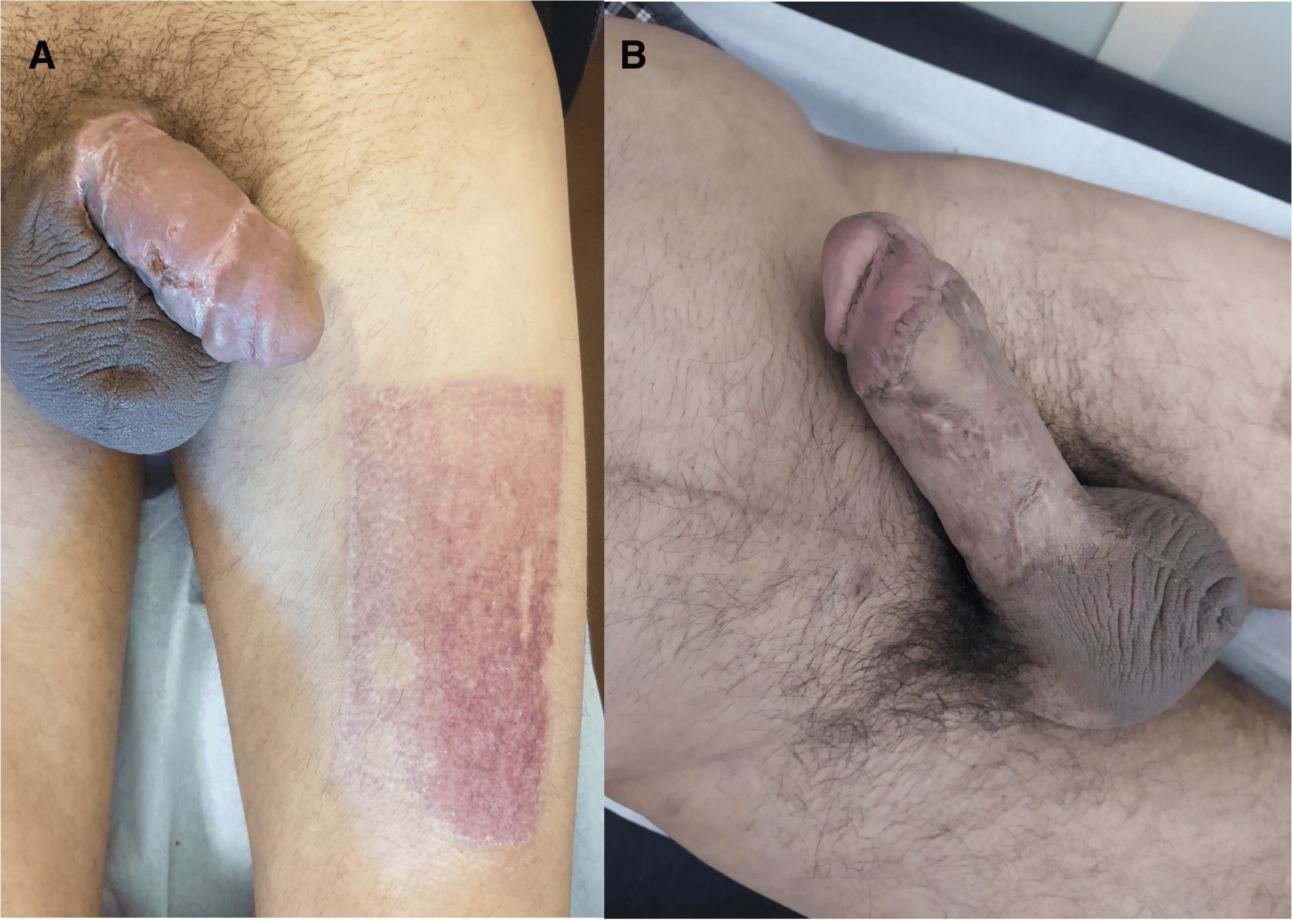
Graft integration: follow-up at one month (A) and 12 months (B).

At one-month follow-up, the patient demonstrated excellent graft healing, satisfactory voiding, and occasional erections (Figure 7A). At six and 12 months, he had maintained erectile function with no need for phosphodiesterase inhibitors (19 points in IIEF-5 score at 12 months) and satisfactory sexual activity and reported no lower urinary tract symptoms. The vasoactive test using intracavernous alprostadil proved a good erectile response (Figure 7B).

## Discussion

Penile strangulation caused by foreign objects requires urgent intervention to prevent irreversible damage. Metallic objects are particularly problematic due to their resistance to cutting tools and potential to cause deep ischemic injury. While various removal techniques have been described, ranging from string methods to orthopedic saws, this case demonstrates the safe and effective use of an angle grinder when other attempts failed [4].

Delayed presentation, commonly due to embarrassment or failed self-removal attempts, significantly increases the risk of tissue ischemia and necrosis [5,9]. In this case, more than 48 hours had elapsed, which typically predicts a poor functional prognosis [2]. Remarkably, despite the risk, this patient experienced complete voiding and sexual function recovery, something rare in high-grade penile injuries.

Multidisciplinary collaboration was essential, involving urologists and plastic surgeons to manage the ischemic injury and perform reconstruction. The outcome clearly exceeded the expectations. Our experience reinforces the need for innovation, caution, and prompt surgical decision-making facing such emergencies.

## Conclusions

The rarity and severity of penile constriction demand prompt recognition, intervention, and decision-making due to the potential for devastating outcomes. This case illustrates that even in a high-grade penile injury with delayed presentation, anatomical salvage and functional recovery are achievable. Multidisciplinary collaboration between urology and reconstructive surgery teams is essential when managing such complex and unusual scenarios.
